# Transmission of Traumatic Lesion

**Published:** 1882-01

**Authors:** 


### CORRESPONDENCE.

### TRANSMISSION OF TRAUMATIC LESION.

Editor Register.--Dear Sir: Mrs. B., when a girl about twelve years of age had a fall, striking upon a piece of broken crockery ware, which made a severe lacerated wound, posteriorly and to the right of the mental process of the inferior maxillary bone, nearly penetrating the floor of the mouth. She married, and eventually became the mother of five children, all of whom have the duplication of cicatrix, and in the same place, as the initial lesion possessed by the mother.

W. Mitchell, Delaware, O.

Remarks. Many years ago, in the Dental Register, we suggested the probable transmission of accidental deformities to offspring. To illustrate, we referred to the fact of a dog, which had been curtailed, (no pun, for he was not a cur dog,) and which was so distinct from all other breeds, in communicating distances, that there was no difficulty in recognizing his offspring. This dog's descendants in many cases lacked caudal appendages. Some wiseacre tried to ridicule the statement, and possibly succeeded; but what will he do with Dr. Mitchell's case?

W.

Waterloo, Dec. 23, 1881.

Editor Dental Register:

Dear Sir:---On December 19th I was called to attend Mrs. W., a lady of eighty-one years, enjoying good health except the discomfort experienced by a malformation of the soft tissues in the region of the left inferior maxilla, which were much enlarged and presented the appearance of a tumor containing fluid. The patient informed me that the condition had existed several years, being at some times much larger than at the present.

Examination evidenced a large quantity of pus confined in the fascia of the surrounding parts, and about the roots of what appeared to be the remains of a first molar. The bicuspids were absent. On applying pressure to the lower part of the face and along the track of the platysma muscle pure pus exuded from the tissue surrounding the root.

Diagnosed the case to be one of latent abscess and proceeded to remove the offending member with a pair of Watling universal forceps, when, to my astonishment, a large mass of something which at first appeared to be bone, was readily lifted from the jaw, which proved to be the remaining roots of the first and second molars, firmly cemented together by bone and calculus. The apex of each tooth had been extensively attacked by caries and that of the first molar had been entirely removed.

By the application of pressure nearly all the pus was removed, after which the parts were thoroughly washed with pure water. I recommended the patient to apply continual pressure to the part with the left hand and rinse the interior frequently with pure water. In three days the discharge had entirely ceased and the swelling subsided.---D. E. P.

